# Application of “immersive contextualization based-learning teaching” mode in the orthopaedic musculoskeletal disorder module of clinical medicine education

**DOI:** 10.1186/s12909-023-04831-y

**Published:** 2023-11-29

**Authors:** Wufeng Cai, Mingke You, Jian Li, Qi Li, Duan Wang, Haoyang Wang

**Affiliations:** 1 Department of Orthopedics, West China Hospital, Orthopedic Research Institute, Sichuan University, Chengdu, China; 2 Department of Engineering, University of Exeter, Exeter, England

**Keywords:** Medical education, Orthopaedic, Immersive contextualization-based learning teaching, Cross-over design

## Abstract

**Objective:**

To evaluate the effect and influence of the “immersive contextualization-based learning” teaching mode (ICBLT) in the orthopaedic musculoskeletal disorder module of clinical medicine education.

**Methods:**

Undergraduate students in five consecutive semesters of clinical medicine in West China Hospital, Sichuan University were enrolled in this study. During the teaching process in each semester, a cross-over design was applied, and students were randomly divided into two classes (Class A and Class B) to receive the designated experimental courses with different routes. After they took the final exams, the scores of the selected chapters (sports injury chapter and osteoarthritis chapter) were extracted to conduct Tests of Between-Subjects Effects. Q-Q plot was drawn to test whether the distribution of the scores follows normal distribution. The part of the feedback questionnaires to assess these two teaching modes were also extracted for comparison.

**Results:**

A total of 441 students were enrolled in this study, among which, Class A teaching route was implemented to 222 students and Class B to the rest 219. The results of Tests of Between-Subjects Effects showed that ICBLT mode could lead to better scores compared to the Lecturing-based learning teaching (LBLT) mode (p < 0.0001). In terms of mastery of practical skills, help to deepen the memory of knowledge and satisfaction with the teaching mode, the ICBLT mode showed better results (p < 0.0001).

**Conclusion:**

ICBLT mode had better potential in helping mastery of practical skills and deepening the memory of knowledge.

**Supplementary Information:**

The online version contains supplementary material available at 10.1186/s12909-023-04831-y.

## Introduction

Orthopaedic musculoskeletal disorder learning is a high-level course in clinical medicine that requires good comprehension and integration of anatomy, histology, pathology, mechanical kinetics and other related fields [1]. The most common teaching plan in medical colleges of China consists of theoretical teaching and bedside internship as the main components, supplemented by problem-based learning (PBL), testing-based learning (TBL) and virtual simulation learning that establish a basic connection between knowledge and clinical practice [2–4]. However, this plan has some limitations, such as the relative isolation of each part and the lack of consideration for the students’ knowledge base, which may hinder their ability to think like experts when they encounter new cases. Contextualization has been proven to have a significant effect on the recall of skills, [5] while the process of clinical reasoning in medicine is much more complex and involves an interaction between knowledge base, experience, and some subtle processes. This is supported by the evidence that students and doctors have different memory patterns for clinical cases [6, 7].

In the traditional learning mode, the teachers or students may play a too dominant or passive role when one is the output and the other is the input, and this kind of complete switch may lead to more ineffective learning or higher learning cost. Inspired by the murder mystery game, [8, 9] we explored the role of the teachers as dungeon masters in the teaching mode, who can guide the students to solve the cases in a more interactive and engaging way. We also considered the feasibility of this approach and tried to reduce the unnecessary cost.

## Method

### Participants

**Fig. 1:**
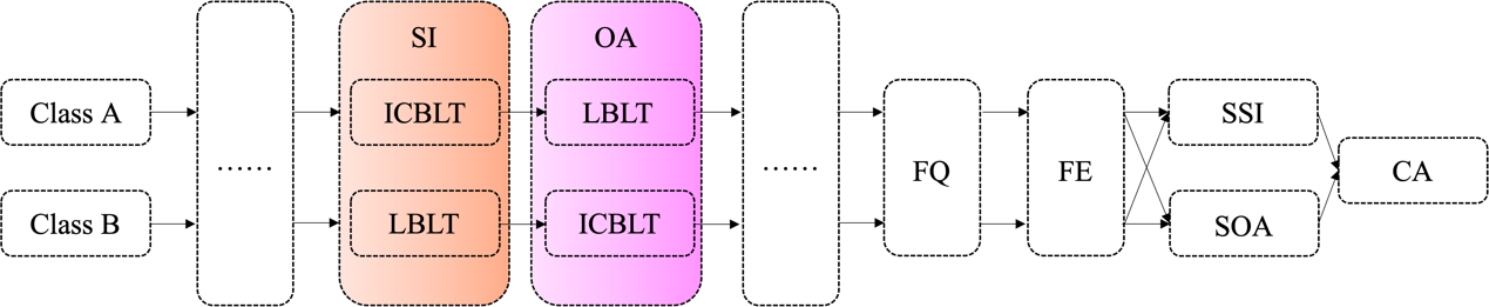
Main flow chart of this research. Abbreviation: ICBLT: immersive contextualization-based learning teaching; LBLT: lecturing based learning teaching; SI: sports injury chapter; OA: osteoarthritis chapter; FQ: feedback of questionnaire; FE: final exam; SSI: scores paired to sports injury; SOA: scores paired to osteoarthritis; CA: correlation ship analysis; ……: other chapters

From March 2018 to Sept 2022, this research enrolled 441 Chinese undergraduate students (in their 4^th^ years) in 5 different semesters, and in each semester, they were randomly divided into two classes that received two separate teaching routes (Fig. 1). The numbers of students in each semester were about 90.

### Study design

We designed the immersive contextualization-based learning teaching (ICBLT) mode as follows. Prior to the official commencement of classes, several student volunteers from higher grade levels who were not enrolled in this course were recruited to participate in the entire process as part of their experience. Then, we invited a real patient with typical symptoms and clear diagnosis related to the corresponding teaching chapter to the class, and the lecturer guided the process of the class. The students obtained the history of illness and positive symptoms through inquiry and physical examination of the patient. The lecturer provided auxiliary clues, such as laboratory and radiological examinations, at appropriate moments. The students were required to make a diagnosis and provide a complete treatment plan based on the evidence they collected, and the lecturer reviewed the whole process and pointed out any missing or incorrect elements that were essential for the correct diagnosis or treatment plan. We also presented small videos of the standard physical examination to obtain relevant positive signs to enhance the memorization. For the lecturing-based learning teaching (LBLT) mode, we presented the knowledge of the disease point by point, including epidemiology, etiology, pathogenesis, clinical manifestations, differential diagnosis, and common treatment methods. We also shared the same supplementary resources material that we provided in the ICBLT mode with the LBLT mode after class.

The final exam had an equal number and weight of questions on the topics of sports injury and osteoarthritis, which were taught using different methods to two classes. The scores for each section were extracted from the exam results and compared using Tests of Between-Subjects Effects.

After the final exam, the students completed feedback questionnaires for the whole teaching module, which included a section to evaluate the two teaching methods. The section asked them to rate the following aspects of each method on a scale of 1 to 5, where 1 was the worst and 5 was the best: how well it built a coherent knowledge framework, how well it taught practical skills, how well it helped them remember the knowledge, and how satisfied they were with it.

To establish content validity, the questionnaire was reviewed by experts who in charge of the education in orthopaedic department of West China Hospital. These experts assessed whether the questions effectively measured the intended constructs, including knowledge framework construction, practical skills, knowledge retention, and teaching satisfaction. Prior to distribution to the main study participants, a pilot test was conducted with a small group of individuals (20 volunteer participants). This pilot test aimed to identify any ambiguities, inconsistencies, or issues with the questionnaire, ensuring its clarity and comprehensibility. The reliability of the questionnaire was determined using statistical methods, specifically Cronbach’s alpha, to measure internal consistency. The responses obtained from the pilot test were analyzed to assess how consistently the questions measured the same constructs. Based on feedback from experts and the results of the pilot test, the questionnaire was revised and refined as needed. These revisions were made to enhance both the validity and reliability of the instrument.

### Data analysis

The data are presented as mean ± standard deviation. Statistical analyses were performed using SPSS 26.0 (IBM Corp. Released 2019. IBM SPSS Statistics for Mac, Version 26.0. Armonk, NY: IBM Corp) with a significance level of P < 0.05. Graphs were generated with GraphPad Prism (version 9.4.1 for Mac, GraphPad Software, San Diego, California USA, www.graphpad.com). The normality of the score distribution was assessed by the Q-Q plot. The independent sample t-test was used to compare the continuous variables between the two groups, and the Chi-square test was used to compare the categorical variables.

### Ethics

Written informed consent was obtained from all participants, and the study protocol was approved by the local research ethics committee of the West China Hospital, Chengdu, China. The study adhered to the relevant guidelines and regulations.

## Results

### Characteristics of the students

This study enrolled 441 students in their fourth years who completed the follow-up. The reason for dropping out was relegation (Table 1). Only the scores of the first attempt of the final exam were collected and analyzed. The students were divided into two groups: 222 students received Class A teaching route and 219 students received another route.

**Table 1:** Characteristics of the enrolled students

Semester	Number of students	Gender (Male/Female)	Age	Scores of final exams	Median	Highest	Lowest	Rate of failure	Rate of excellent
2018	88	40/48	21.90 ± 0.92	80.74 ± 5.71	81	92	52	2.2%	1.1%
2019	90	43/47	22.01 ± 0.85	80.12 ± 4.87	81	92	60	0	1.1%
2020	84	39/45	22.03 ± 0.82	86.75 ± 9.94	88	94	79	0	13.1%
2021	90	49/41	21.68 ± 0.82	83.30 ± 3.54	84	92	75	0	2.2%
2022	89	45/44	21.94 ± 0.83	81.34 ± 4.04	82	90	71	0	2.2%

### Related scores of the final exam

**Fig. 2:**
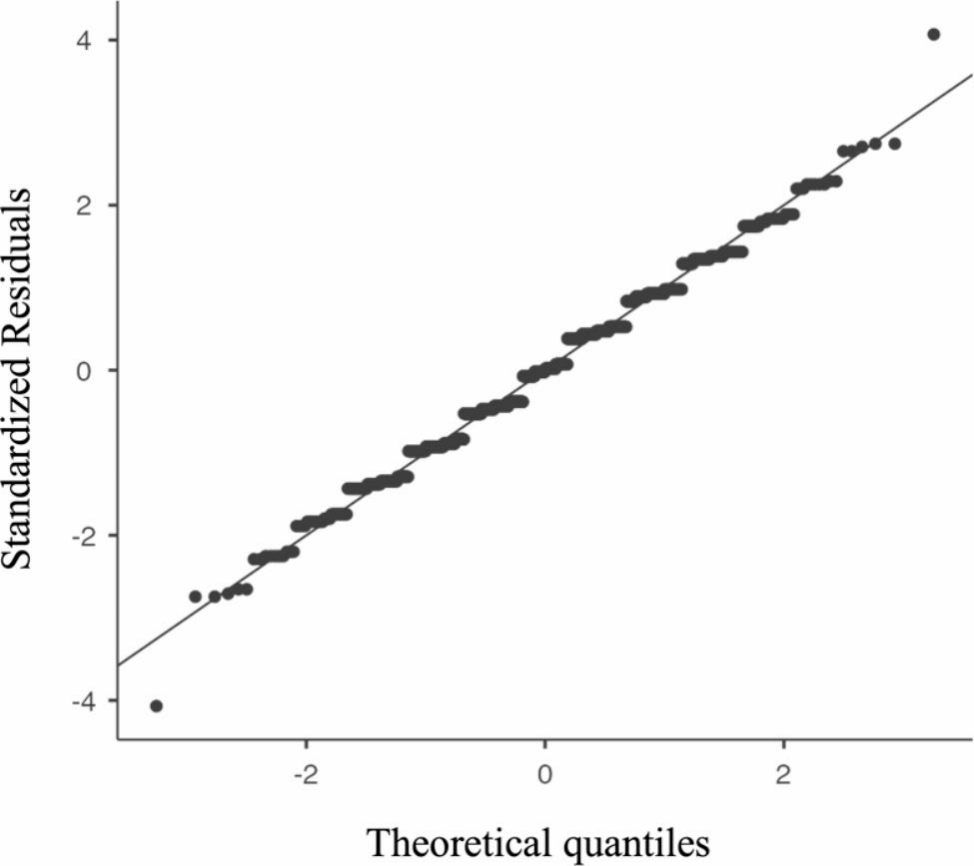
Q-Q Plot of the score outcomes related to the sports injury chapter and OA chapter

The Q-Q Plot showed that the scores followed a normal distribution, as they were close to the diagonal line (Fig. 2).

**Fig. 3:**
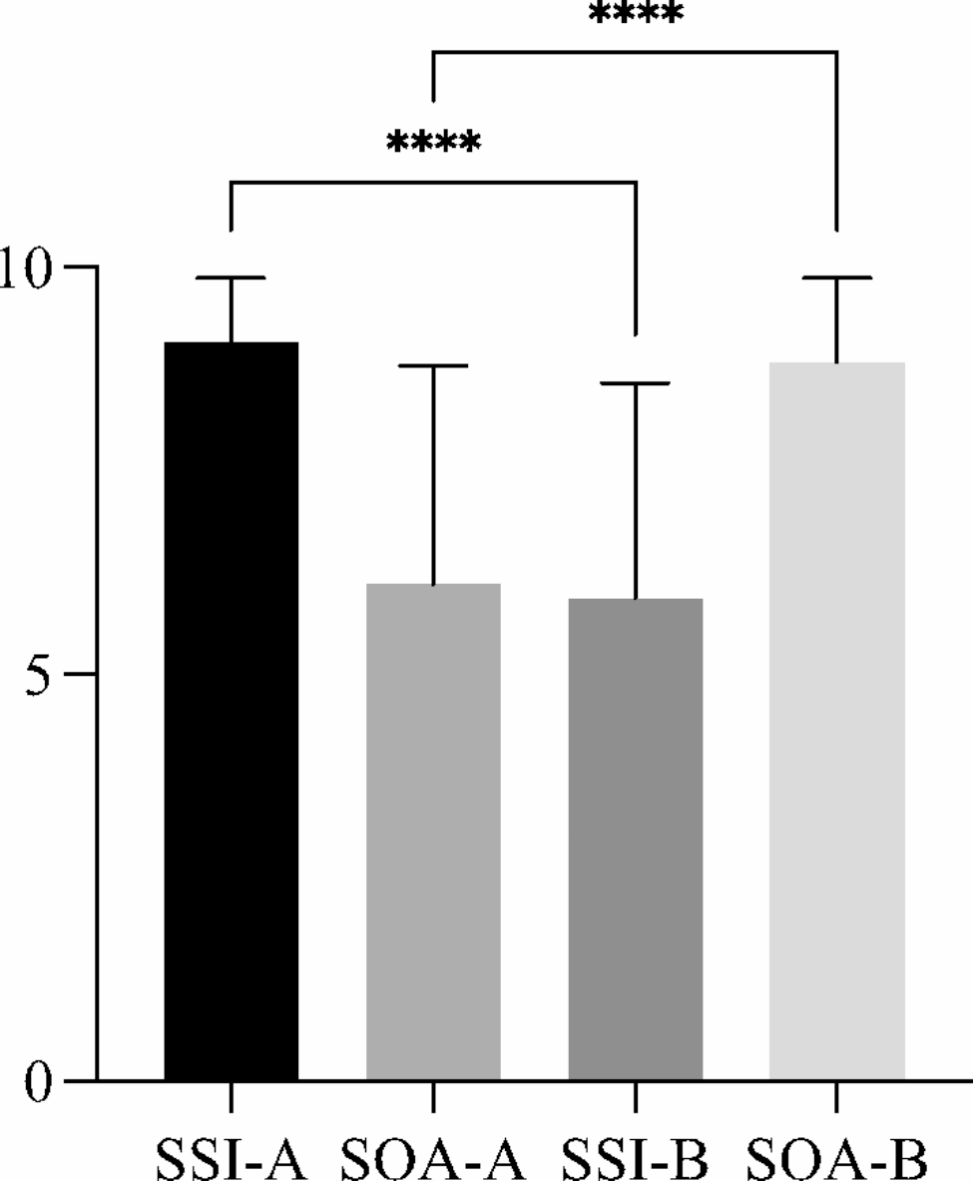
Extracted scores of sports injury and osteoarthritis parts from exam results. SSI-A: Score of sports injury of Class A; SOA-A: Score of osteoarthritis of Class A; SSI-B: Score of sports injury of Class B; SOA-A: Score of osteoarthritis of Class B; ^****^: *p* < 0.0001

The results indicated that the ICBLT mode was more effective than the LBLT mode in improving the scores (Table 2; Fig. 3). The teaching routes and the baseline of the students did not have a significant effect on the scores (Table 2).

**Table 2:** Extracted scores of sports injury and osteoarthritis parts from exam results

	Class A	Class B
Sports injury	9.08 ± 0.79	5.93 ± 2.64
Osteoarthritis	6.10 ± 2.68	8.83 ± 1.03

### Feedback questionnaires

**Fig. 4:**
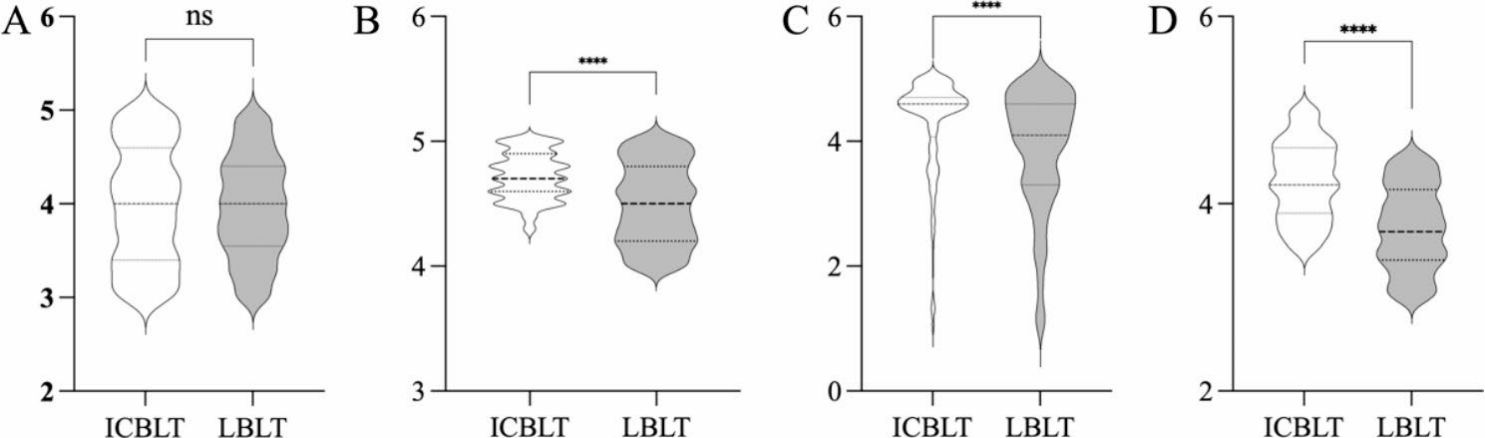
Violin plot of the results of (A) integrity of the knowledge framework construction; (B) mastery of practical skills; (C) help to deepen the memory of knowledge; (D) satisfaction with the teaching mode; ICBLT: immersive contextualization-based learning teaching; LBLT: lecturing-based learning teaching; ^****^: *p* < 0.0001

The feedback questionnaires (Supplementary material 1) compared the Interactive Case-Based Learning and Teaching (ICBLT) mode with the Lecture-Based Learning and Teaching (LBLT) mode across four key aspects: knowledge framework construction, practical skills, knowledge retention, and teaching satisfaction (Fig. 4). Questions 1 and 2, as well as questions 3 and 4, were treated as separate pairs, with question 4 designed as a reverse item to evaluate the questionnaire’s reliability. The overall Cronbach’s α coefficient for the questionnaire was 0.775, indicating a high level of reliability. During the analysis, the scores of questions 1 and 2, as well as questions 3 and 4, were combined and presented as mean ± SD. Both modes performed well in terms of the first aspect, suggesting a solid foundation in knowledge framework construction. However, the ICBLT mode outperformed the LBLT mode in the other three aspects, demonstrating superior proficiency in practical skills, a deeper retention of knowledge, and higher satisfaction with the teaching approach (p < 0.0001).

## Discussion

### How to establish the modified ICBLT mode

The term “modified” refers to the adaptation of the teaching mode to the feasibility, patients’ cooperation, and students’ experience. The COVID-19 pandemic has posed challenges to offline education [10–12]. Orthopaedic education could be worse affected by the cancellation or postponement of elective surgeries, which leads to a drastic reduction of orthopaedic case volume [13]. Therefore, exploring the possibility of replacing face-to-face education with online education has become a trend and has been proven effective [14–17]. However, these efforts may be a kind of compromise, as the final step is always to communicate with the patients face to face. Moreover, the course does not aim to train all the participants to be orthopaedic surgeons in the future. Therefore, feasibility should be the primary consideration.

For feasibility, the LBLT mode could be used for most classes, as it also showed good results in knowledge framework construction compared to the ICBLT mode, according to our findings. The ICBLT mode could be reserved for some classes that require more practical skills and patient interaction. We suggest selecting common diseases with typical positive signs and patients with a hospital stay of about one week. Moreover, we recommend setting up a teaching ward with the necessary equipment, such as a laptop, a screen, a projector, virtual human models, and a sickbed.

To ensure patients’ cooperation, we applied for a special fund to cover the extra cost of hospital stay (usually one more day) for the patients who participated in the teaching. This way, we did not compromise the normal treatment time of the patients or charge them extra fees. Previous research has reported that repeated physical examination may cause pain and discomfort to the patients, but it may also enhance the long-term retention and application of knowledge for the students [18–20]. The physical examination process was modified as follows: one or two students examined the patients first, then practiced on the virtual human models, and finally watched the standard videos.

The students’ learning experience was enhanced by the ICBLT mode, which integrated problem-based learning. The ICBLT mode provided some focus points during the illness query process and some clues to guide the correct direction. This helped the students who had not yet developed clinical thinking to complete the whole process with more initiative and less difficulty [21]. The purpose of this teaching mode is to help the students construct an immersive context for their first encounter with these common diseases, which can enhance their memory and interest, rather than to test their clinical abilities or demand too much preparation before the class.

### Contextualization in memory construction

The ICBLT mode is a type of case-based learning (CBL) that allows more control over the learning process. The case-based method has many advantages, such as fostering self-directed learning, clinical reasoning, clinical problem solving, and decision making. It also provides students with repeated experiences in class and helps them focus on the complexity of clinical care [22, 23]. The real patients, the vivid voice, the complete illness history, the positive signs related to the diseases, and the context we create, all contribute to a complete story in the students’ mind. However, this depends on the students’ ability to connect these fragmentary materials. For novices, without adequate support, their problem-solving skills are very weak [24]. The lecturer’s timely guidance is crucial in this case, and his role can be inspired by the dungeon master of the murder mystery game [25].

Traditional CBL has been criticized for affecting both teachers’ and students’ experience without adequate preparation [26]. To make the clues more effective and less intrusive, we will recruit several student volunteers who are not enrolled in this course to experience the whole process before the class. Based on their knowledge base, they will help us determine the appropriate clues to provide during the class.

### Ethic consideration

In fact, this process often takes place within a dedicated teaching room in a hospital skills center. This room is equipped with distinct areas, including a consultation area, a patient bed area, an examination area, and a teaching area. It provides students with a comprehensive experiential journey, covering various stages from initial diagnosis, admission, physical examination, laboratory tests, to treatment. For patients, this process closely resembles bedside teaching, and they are explicitly informed about the informed consent process and the protection of their privacy before participating.

In this structured teaching environment, students gain a holistic understanding of patient care, clinical procedures, and decision-making while respecting ethical considerations [27]. The inclusion of a specific room layout further enhances the realism of the educational experience. These measures not only contribute to the professional development of students but also prioritize patient well-being and privacy, emphasizing the ethical principles that guide medical education and practice. Certainly, it’s important to note that ethical considerations are considerably more complex and demand careful attention [28].

### Limitation

This study has some limitations. The teaching mode we proposed is only suitable for medical colleges that have affiliated teaching hospitals. Also, the selection of diseases for the course is restricted by the patients’ cooperation and the potential risks to them. Furthermore, the course arrangement requires some preparation and rehearsal. These challenges can be overcome by optimizing the curriculum structure and applying for relevant educational funds.

## Conclusion

Both modes were effective in building a knowledge framework, but the ICBLT mode had more advantages in enhancing practical skills and knowledge retention, which resulted in better impressions and scores.

### Electronic supplementary material

Below is the link to the electronic supplementary material.

Supplementary Material 1

## Data Availability

The relevant original data of participants can be obtained from Haoyang Wang, wanghaoyang-scu@163.com.
