# Supplementary material for: Application of “immersive contextualization based-learning teaching” mode in the orthopaedic musculoskeletal disorder module of clinical medicine education
Source: BMC Med Educ. 2023 Nov 29;23:906. doi: 10.1186/s12909-023-04831-y (PMC10687819; doi:10.1186/s12909-023-04831-y)
Supplement: Supplementary file 1 — Supplementary Material 1 [file 12909_2023_4831_MOESM1_ESM.docx]

Supplementary material 1

**Feedback Questionnaire**

Introduction:

Thank you for participating in this survey. Your feedback will help us better understand the effectiveness of two teaching modes: Immersive Contextualization-Based Learning and Lecturing-Based Learning. Please answer the following questions honestly and to the best of your knowledge and experience. Meanwhile the feedback you provide is not related to your final exam results. 1: Strongly Disagree; 5: Strongly Agree.

- Name (Optional):
- Age:
- Gender:

***Section 1: Knowledge Framework Construction***

**Immersive Contextualization-Based Learning**

1.The immersive contextualization-based learning mode helped me understand complex concepts better.


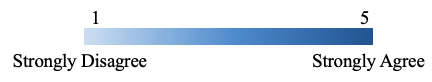


2.This teaching mode encouraged me to connect knowledge to real-world situations.


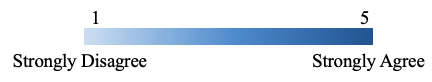


**Lecturing-Based Learning**

3.The lecturing-based learning mode effectively conveyed information.


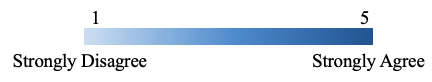


4.I found it challenging to relate the lecture content to real-life applications.


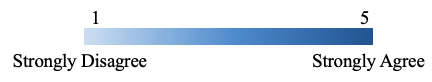


***Section 2: Practical Skills***

**Immersive Contextualization-Based Learning**

5.The immersive contextualization-based learning mode helped me develop practical skills.


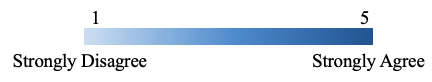


**Lecturing-Based Learning**

6.The lecturing-based learning mode helped me develop practical skills.


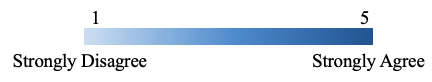


***Section 3: Knowledge Retention***

7.I feel that I retained more knowledge from the immersive contextualization-based learning mode.


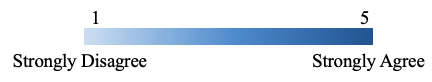


8.I feel that I retained more knowledge from the lecturing-based learning mode.


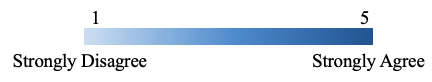


***Section 4: Teaching Satisfaction***

9.I found the immersive contextualization-based learning mode more engaging and enjoyable.


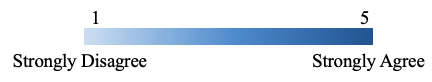


10.I found the lecturing-based learning mode more engaging and enjoyable.


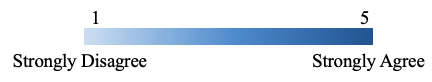


Additional Comments:

Please share any additional comments or feedback about your experience with both teaching modes.

Thank you for your participation! Your feedback will be valuable for our study on teaching effectiveness.
